# Structured Light Three-Dimensional Measurement Based on Machine Learning

**DOI:** 10.3390/s19143229

**Published:** 2019-07-23

**Authors:** Chuqian Zhong, Zhan Gao, Xu Wang, Shuangyun Shao, Chenjia Gao

**Affiliations:** Key Laboratory of Luminescence and Optical Information of Ministry of Education, Beijing Jiaotong University, Beijing 100044, China

**Keywords:** structured light, 3D measurement, machine learning, LSSVM

## Abstract

The three-dimensional measurement of structured light is commonly used and has widespread applications in many industries. In this study, machine learning is used for structured light 3D measurement to recover the phase distribution of the measured object by employing two machine learning models. Without phase shift, the measurement operational complexity and computation time decline renders real-time measurement possible. Finally, a grating-based structured light measurement system is constructed, and machine learning is used to recover the phase. The calculated phase of distribution is wrapped in only one dimension and not in two dimensions, as in other methods. The measurement error is observed to be under 1%.

## 1. Introduction

Structured light three-dimensional measurement technology is an active optical measurement technology with the characteristics of small volume, low price, and easy installation and maintenance [1]. Therefore, this technology exhibits a wide range of applications in actual industrial fields, such as vision inspection [2,3], face profilometry [4,5,6], and robots [7,8,9]. The light source projects the structured light with a certain shape pattern, such as a light spot, a light strip or a light surface structure, and the structured pattern reflected by the object is obtained using an image sensor, such as a charge-coupled device (CCD). The grating pattern is a structured light pattern, which is mostly used in structured light measurement, as it does not require scanning the whole surface unlike the point-structured light and line-structured light to obtain three-dimensional information. This is a remarkable advantage of surface structured light technology, which significantly improves the computational efficiency and renders real-time three-dimensional measurement possible. Phase extraction, analysis, and unwrapping technologies are the research hotspots in grating projection 3D measurements. For example, the phase-shifting method [10] and Fourier transform method [11] have significantly promoted the development of grating projection measurement technology. The phase-shifting method uses a phase shifter, such as the piezoelectric ceramic phase shifter, to generate phase shifts, each time moving the same phase shift. According to how the phase shift amount is determined, the phase-shifting method can be divided into two categories, including the fixed step length phase-shift method and equal step phase-shift method. The use of Fourier transforms in three-dimensional structured light measurements to recover the phase was proposed by Takeda M et al. in 1982 [11]. Later, Claude Roddier and Francois Roddier applied the two-dimensional Fourier transform for full-field phase extraction [12]. Their principle was to obtain the phase by filtering out the alternating component of the signal in the spectrum. The shape and bandwidth of the filter would influence phase recovery results. The rectangle window usually performs well while its computational complexity is higher than other filters [13]. In addition, some techniques have also been proposed to enhance the performance of Fourier transform profilometry. For example, a grating π phase shifting technique can enlarge the measurable slope of height variation significantly [14]; the principal component analysis is used to extract the phase of the carrier from the first dominant component [15]; the binocular stereo vision and two image pairs, original image pairs and fringe image pairs, is used to restructure 3D shape and a coarse disparity map is employed to calculate the phase distribution [16].

Usually, the extracted phase distribution is wrapped in two dimensions. The two-dimensional phase unwrapping is a complex and abstract technology, which can be organized into two main classes: path-following algorithm and minimum-norm algorithm [17]. The results of the path-following algorithm can be influenced by the integration path, and discovering the appropriate path is a complicated process. The distribution unwrapped by the minimum-norm algorithm is a fitted distribution, which demonstrates a deviation compared to the real distribution. Meanwhile, during practical measurements, various factors, such as the noise and shadow, may lead to unreliable phase distribution and errors in unwrapping.

Machine learning is one of the key technologies in the field of artificial intelligence. It is very popular in many fields, such as computer vision [18,19], pattern recognition [20,21], and aerospace [22]. Machine learning includes many methods, such as back-propagation neural networks [23], support vector machines [24], and convolutional neural networks [25]. The principle of machine learning is to acquire knowledge using training samples and demonstrate an optimal performance in classification and regression. In regression, to obtain the function between the inputs and outputs, machine learning can be used to train the input and output samples and obtain the model as the required function. 

In this study, the machine learning method was used to recover the phase from the structured light pattern, and the least squares support vector machine was considered to train the models. This method eliminated the phase-shifting process, and therefore, it could be used in real-time 3D measurement. On the other hand, the calculated phase was wrapped in only one dimension, which means two-dimensional phase unwrapping was not required any more, and this simplified the process of phase unwrapping greatly. Two models were trained to satisfy the condition of the measuring experiment, because training only one model to cover all the phases of the initial phase of the light intensity signal was difficult. The three-dimensional measurement was successful with a measuring error of under 1%, which indicates that the machine learning models can be employed in numerous applications related to structured light 3D measurement. 

## 2. Methods

### 2.1. Structured Light Three-Dimensional Measurement

**Figure 1:**
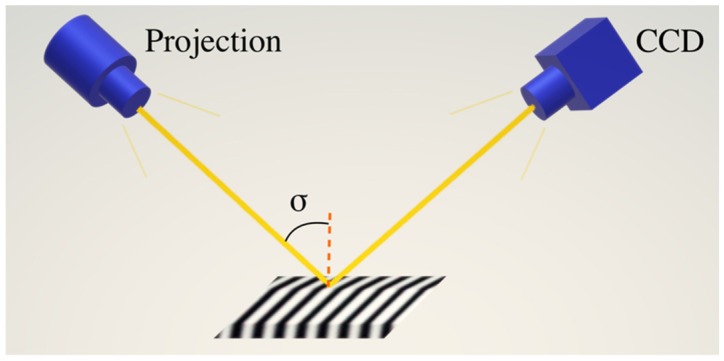
Optical path diagram of surface measured by a structured light measuring system.

The schematic diagram of structured light three-dimensional measurement is shown in Figure 1. The grating fringes with sinusoidal phase are projected onto the surface of the measured object. The deformation of the grating pattern collected by the camera is different due to the varied heights of the surface to be measured. This also implies that the phase of the grating image is no longer uniformly distributed. The phase distribution of the grating image is calculated using the phase extraction and unwrapping technologies and subtracted from the phase of the background reference plane to obtain the real phase of the object surface. The surface of the measured object is finally obtained using the function between the phase and height.

The deformed structured light intensity received by the CCD is defined as follows:

(1) I(x,y)=A(x,y)+B(x,y)cos(φ(x,y)+θ),

where I(x,y) is the intensity of the grating fringe pattern, A(x,y) is the background light intensity, B(x,y) is the amplitude of the grating field, and θ is the additional phase. The wrapped phase of the grating fringe pattern can be extracted using the phase extraction algorithm. The phase obtained in Equation (1) is then unwrapped and the phase of the measured surface φ(x,y)+θ is obtained. The relative phase distribution of the measured object surface $\varphi_{h}\left(x,y\right)$ is defined as follows:

(2) $$\varphi_{h}\left(x,y\right)=\varphi \left(x,y\right)-\varphi_{r}\left(x,y\right),$$

where $\varphi_{r}\left(x,y\right)$ is the phase distribution of the reference plane. The function between the height H(x,y) and phase is linear according to the 3D phase measurement principle:

(3) $$H\left(x,y\right)=\frac{p_{0}}{2\pi tan\sigma }\varphi_{h}\left(x,y\right),$$

where $p_{0}$ is the grating frequency, and σ is the angle of the incident light.

Using Equation (3), the height of each point on an object can be calculated. However, in practical measurements, the function between the phase distribution and height is mostly non-linear, because of the divergence of incident surface structured light and aberration of the camera lens. To simplify the measurement process and obtain a precise measuring result, we need to calibrate the relationship between the height and phase of the object surface, and we generally use polynomial fitting to achieve this calibration.

The relationship between the height and phase of the object surface can be expressed as follows:

(4) $$h\left(x,y\right)=\sum_{i=0}^{n}a_{n}\varphi_{n}\left(x,y\right),$$

where n is the order of the polynomial fitting, and $a_{n}$ are the coefficients of polynomial fitting. 

The purpose of the calibration process is to calculate the phase distribution $\varphi_{n}\left(x,y\right)$ of every calibration plane with sinusoidal grating fringes and record the depth of these planes $h_{n}$. The optimal polynomial coefficients of each point in the light field $a_{n}$ are obtained using the polynomial fitting algorithm. To ensure the accuracy of the results corresponding to the measured surface, the measured object must be placed in the calibration interval.

### 2.2. Least Squares Support Vector Machine Regression

Statistical machine learning is a machine learning technology that has been widely used in recent years. It uses the statistical theories to train an optimal model from the input space to the output feature space. 

The support vector machine (SVM) is one of the representative methods of statistical machine learning and was proposed by Vapnik in 1963. This method trains a hyperplane to represent the nonlinear function between the inputs and outputs [24]. The least squares support vector machine (LSSVM) was developed from SVM and first proposed by Suykens in 1999 [26]. The LSSVM changes the inequality constraints into equality constraints in SVM and changes the empirical risk from the first order to second order, as defined in Equation (6). Therefore, training the LSSVM model involves solving a linear equations system. This aspect is a major advantage of LSSVM, as it reduces the computational complexity significantly.

The algorithm of regression using the LSSVM model is introduced as follows:

Set the training data set as follows: $\left(x_{i},y_{i}\right),\;i=1,\;2,\ldots ,l,x_{i}\in R,y_{i}\in R$, where $x_{i}$ are the input samples, and $y_{i}$ are the output samples.

The expression of hyperplane in the feature space is as follows:

(5) $$y\left(x\right)=\omega^{T}\phi \left(x\right)+b.$$

According to Equation (5), the input samples are mapped nonlinearly into the high-dimensional feature space. According to the principle of structural risk minimization, the linear regression problems in the high-dimensional feature space can be expressed as a constrained optimization problem as follows:

(6) $$\underset{\omega ,b,e}{\min}J\left(\omega ,e\right)=\frac{1}{2}\omega^{T}\omega +\frac{C}{2}\sum_{i=1}^{l}e_{i}{}^{2},$$

(7) $$s.t.\;y_{i}=\omega^{T}\phi \left(x\right)+b+e_{i},i=1,2,\ldots ,l.$$

To solve the optimization problem defined in Equation (8), we use the Lagrange function to transform the constrained optimization problem into an unconstrained optimization problem as follows:

(8) $$L\left(\omega ,b,e,\alpha \right)=J\left(\omega ,e\right)-\sum_{i=1}^{l}\alpha_{i}\left[\omega^{T}\phi \left(x\right)+b+e_{i}-y_{i}\right].$$

According to the Karush–Kuhn–Tucker conditions,

(9) $$\{\begin{array}{c} \frac{\partial L}{\partial \omega }=0\to \omega =\sum_{i=1}^{l}\alpha_{i}\phi \left(x_{i}\right)\\ \frac{\partial L}{\partial b}=0\to \sum_{i=1}^{l}\alpha_{i}=0\\ \frac{\partial L}{\partial e_{i}}=0\to \alpha_{i}=Ce_{i}\\ \frac{\partial L}{\partial \alpha_{i}}=0\to \omega^{T}\phi \left(x_{i}\right)+b+e_{i}-y_{i}=0 \end{array}.$$

By eliminating $e_{i}$ and ω from Equation (9), we can obtain the following:

(10) $$\left[\begin{array}{cc} 0 & E^{T}\\ E & Q+C^{-1}I \end{array}\right]\left[\begin{array}{c} b\\ \alpha \end{array}\right]=\left[\begin{array}{c} 0\\ y \end{array}\right],$$

where $y={\left(y_{1},y_{2},\ldots ,y_{l}\right)}^{T}$, $E={\left(1,1,\ldots ,1\right)}^{T}$, $\alpha ={\left(\alpha_{1},\alpha_{2},\ldots ,\alpha_{l}\right)}^{T}$, $Q_{ij}=\left(\phi \left(x_{i}\right)\cdot \phi \left(x_{j}\right)\right)=k\left(x_{i},x_{j}\right),i,j=1,2,\ldots l$ and I is an identity matrix.

Therefore, the outputs y(x) of the LSSVM model are defined as follows:

(11) $$y\left(x\right)=\sum_{i=1}^{l}\alpha_{i}k\left(x,x_{i}\right)+b.$$

## 3. Experiment and Results

**Figure 2:**
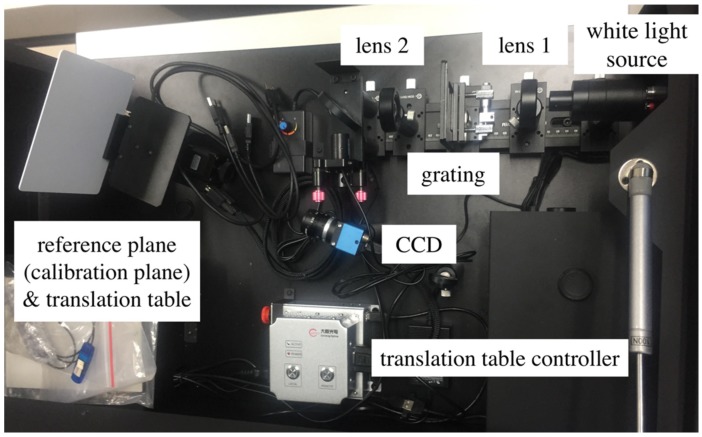
Structured light 3D measurement system.

A structured light three-dimensional measurement was conducted using an optical sensing 3D shape measurement instrument GCS-SWCL from DAHENG Company. The experimental light path is shown in Figure 2. The light was emitted from the white light source, which then passed through lens 1 and grating and produced a sinusoidal structured light. The structured light was then collimated by lens 2 and projected to the measured object. Finally, the CCD captured the fringe pattern of the measured object.

### 3.1. Algorithms and Models

The primary notion of the phase extraction technology based on machine learning is to train the function between the input light intensity signal and corresponding output phase. Firstly, the training data were obtained as follows: the structured light intensities in one dimension with different phases were considered as the input samples, and corresponding phases were considered as the output samples. These samples were trained using the machine learning method to obtain a multiple-inputs and multiple-outputs regression model. The light intensity signals from the structured light 3D measurement were collected by the CCD. Normalized intensity signals were used as test input samples, and phase distributions of the test intensities were considered as the outputs. Finally, the depth information of the object was calculated using the phase of the test light intensities.

The range of the training set could be estimated directly from the structured light fringe pattern. Moreover, the spatial frequency from one line in the pattern could be obtained using traditional methods, such as the Fourier transform, to estimate the range of the full field’s spatial frequency. In this experiment, the range of the training set was estimated by the spatial frequency of one line in a calibration plane calculated using the Fourier transform (please refer to the details of the range of training sets in Model 1 and Model 2 in the following). 

The model trained by the LSSVM can extract phase in one dimension of the pattern. The initial phase of the training input set must be in the range of 0–2π; however, it is difficult for only one model to include the whole range of initial phase, because numerous samples utilize considerable training calculation time. Therefore, our study required two models to complete the measurement jointly, which could cover the range of up to 2π in the direction that was vertical to the phase extraction’s direction.

For example, the training samples of the two models used in the planar surface measurement were as follows:

Model 1: The training samples of input intensity signals are $X\left(x\right)=\cos\left(v_{i}\cdot x+\varphi_{0i}^{\prime }\right)$, where the range of $v_{i}$ is from 15–20, the range of $\varphi_{0i}^{\prime }$ is from 0–1.7π, and the range of x is from 0–0.49. The training output samples are defined as the corresponding phases $Y\left(x\right)=v_{i}\cdot x+\varphi_{0i}^{\prime }$.

Model 2: The training samples of input intensity signals are $X\left(x\right)=\cos\left(v_{i}\cdot x+\varphi_{0i}^{\prime }\right)$, where the range of $v_{i}$ is from 15–20, the range of $\varphi_{0i}^{\prime }$ is from 1.5π–2.5π, and the range of x is from 0–0.49. The training output samples are defined as the corresponding phases $Y\left(x\right)=v_{i}\cdot x+\varphi_{0i}^{\prime }$.

The specific phase extraction algorithm is defined as follows:

The pattern is segmented to several parts. Each part includes 50 lines. The first part includes the first to the 50th line.

For the selection of the initial model, if normalized light intensity $I\left(x_{1},y_{1}\right)>0$, Model 1 is used. Otherwise, Model 2 is used.

The same model is used for every 50 lines. If the initial phase of a line in 50 rows exceeds the specified interval, which is [0.5, 4.5] for Model 1 and [4.5, 7.5] for Model 2, the line is recorded. Then, the next part is initiated from the line recorded in the previous part. If the initial phase of each line in the part is within the prescribed interval, the same model is used to calculate the next part.

### 3.2. Calibration

**Figure 3:**
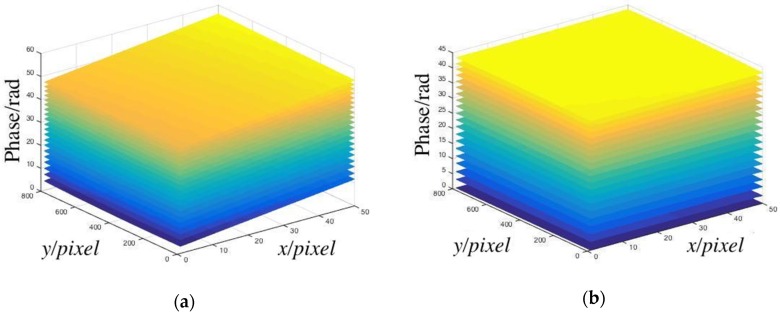
(**a**) Phases of calibration planes; (**b**) relative phases of calibration planes.

The calibration process must be completed before the measurement. The 2-line/mm sinusoidal grating was placed in the device. The white light source was opened, and the CCD was regulated to a suitable location so that the calibration plane with grating fringes could fill the complete CCD image plane. The translation table was controlled by a computer and moved from 4–80 mm of the grating, and the pictures were captured, when the translation table moved by 4 mm each time. After each movement, the background plate was photographed and the phase was recovered by the LSSVM models. The phase recovering results are shown in Figure 3a.

The phase distribution of each image was subtracted from the phase of the first background plane to obtain the relative phase, as shown in Figure 3b.

The phase and height of each pixel in the relative phases of the calibration planes data cube were extracted, and the function between the phase and height of each pixel was fitted using the five-order polynomial fitting. The coefficients of the five-order polynomials were saved into the coefficient data cube.

### 3.3. Measurement and Results

#### 3.3.1. Planar Surface

**Figure 4:**
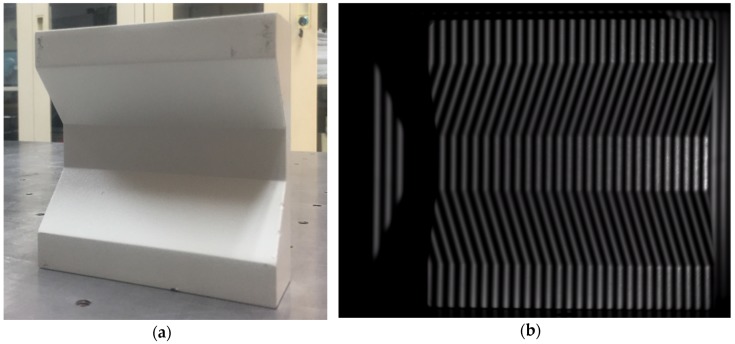
(**a**) Measured object; (**b**) grating fringe pattern of the measured object.

After the calibration was completed, the measured object was placed in the calibration interval. The measured object is shown in Figure 4a.

The measurement experiment was then initiated. The grating structured light fringe patterns of the measured object were captured by the CCD, as shown in Figure 4b.

**Figure 5:**
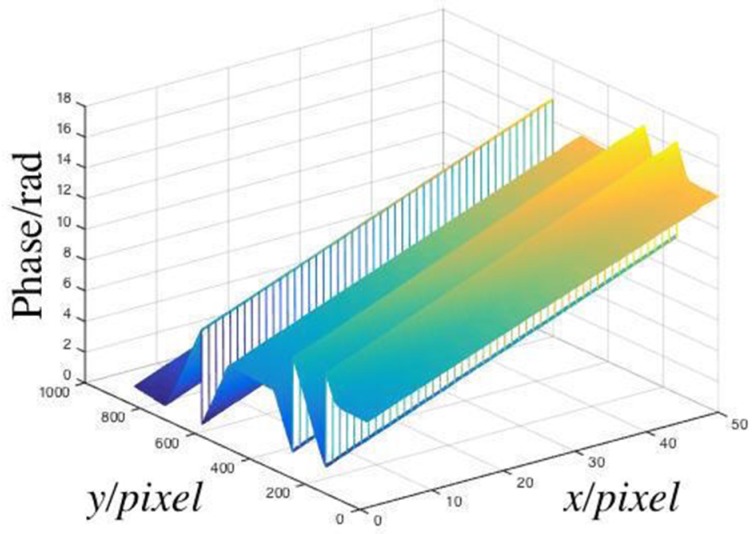
Phase of the measured object.

We considered the area of the lines 100–799 and columns 480–529 to calculate the phase distribution, which were arbitrary chosen. The full-field phase distribution was obtained using the models we trained, and this distribution is shown in Figure 5.

**Figure 6:**
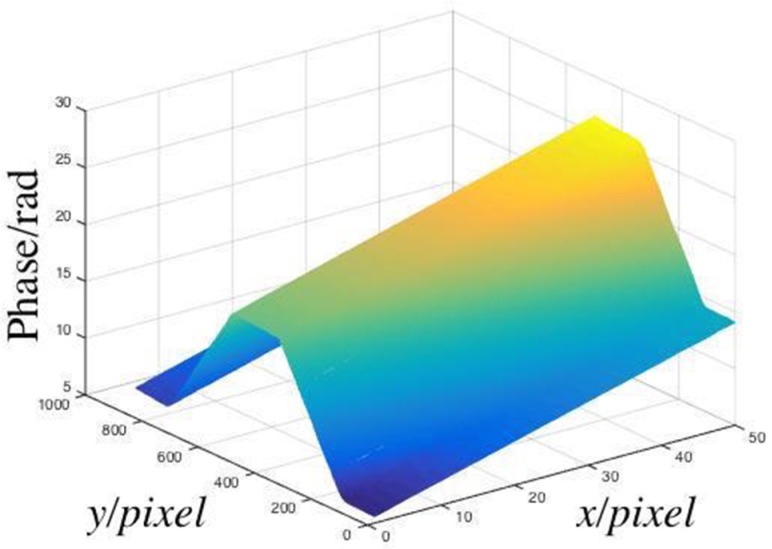
Unwrapped phase of the measured object.

The machine learning models were used to recover the phase distribution in the x-direction, which was unwrapped. However, in the y-direction, the phase distribution was wrapped. Therefore, the phase must be unwrapped in the y-axis. The unwrapping phase distribution is shown in Figure 6.

**Figure 7:**
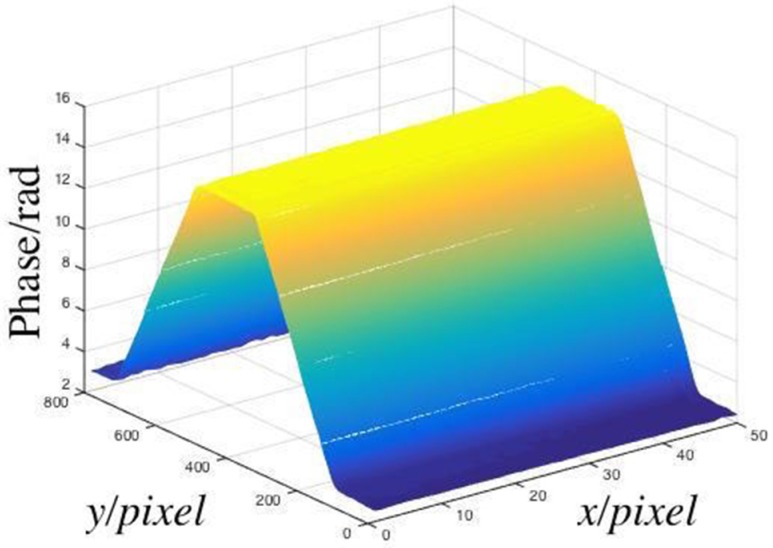
Relative phase of the measured object.

After subtracting the background phase, the relative phase was obtained, which is shown in Figure 7.

**Figure 8:**
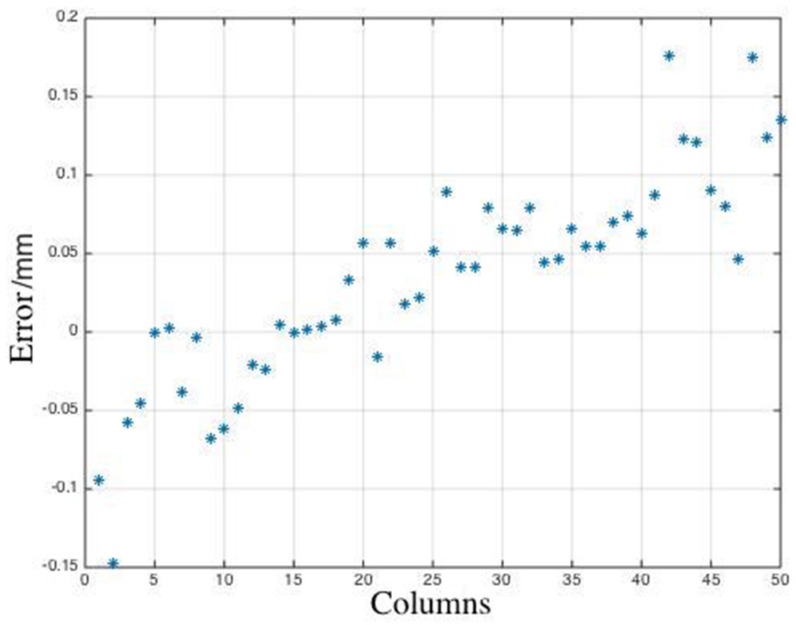
Measurement errors corresponding to columns 480–529.

Finally, the depth of the object was calculated using the calibration coefficients, the height distribution was fit using a certain fitting algorithm, and the height difference between the top and bottom surfaces was obtained (the theoretical value is 20 mm). The measurement errors corresponding to columns 480–529 are shown in Figure 8. 

#### 3.3.2. Curved Surface

To validate that our method could be used in a large range of applications, the phase distributions corresponding to two different curved surfaces were measured.

##### Simple Curved Surface

**Figure 9:**
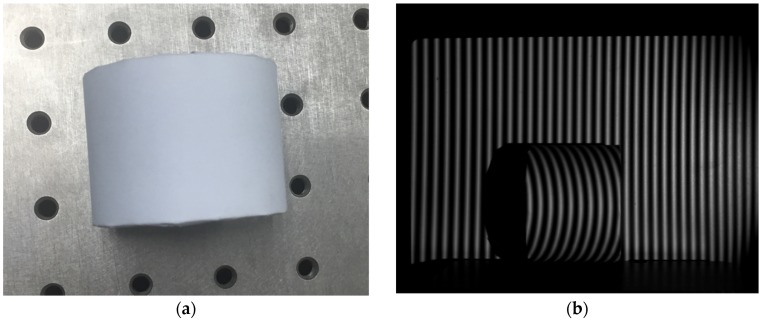
(**a**) Measured cylinder; (**b**) Measured cylinder’s structured light fringe pattern.

The cylinder is a typical simple curved surface, which is measured frequently in many industrial situations. Owing to its linear phase distribution on the *x*-axis, the model is the same as that of the plane surface measurement. However, the noise in the cylinder’s fringe pattern is different from that of the planar object, and the performance of the model to adapt the pattern is different as well. Hence, the specified intervals of the initial phase for Model 1 and Model 2 were changed to [1, 4.2] and [4.5, 7.5], respectively. The measured cylinder is shown in Figure 9a, and its structured light fringe pattern is shown in Figure 9b.

**Figure 10:**
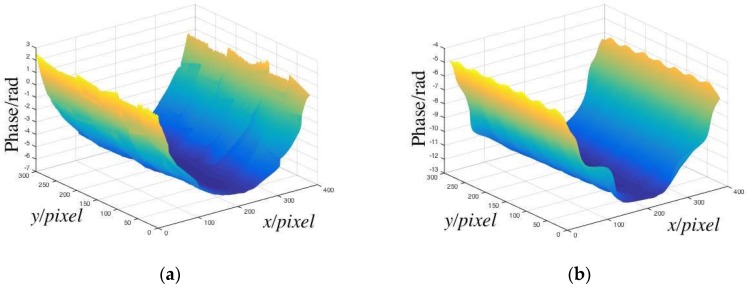
(**a**) Cylinder’s phase distribution calculated by machine learning; (**b**) cylinder’s phase distribution calculated by FFT.

The phase distribution calculated by the machine learning model is shown in Figure 10a. As the CCD was not capable of capturing the entire measured surface, it was difficult to measure the height difference between the top and bottom of the cylinder. Meanwhile, we calculated the phase distribution with the algorithm of fast Fourier transform(FFT) techniques presented by Mitsuo Takeda for comparison [27]. Its principle is introduced briefly as follows: 

The intensity of the fringe pattern can be written as

(12) $$I\left(x,y\right)=a\left(x,y\right)+b\left(x,y\right)\cos\left(\Phi \left(x,y\right)+2\pi f_{0}x\right).$$

where the phase Φ(x,y) includes the desired information. a(x,y) and b(x,y) represent variations caused from the nonuniform light reflection or transmission by the measured object, and $f_{0}$ is the spatial-carrier frequency. It can be further written as

(13) $$I\left(x,y\right)=a\left(x,y\right)+c\left(x,y\right)e^{j2\pi f_{0}x}+c^{*}\left(x,y\right)e^{-j2\pi f_{0}x}.$$

where $c\left(x,y\right)=\frac{1}{2}b\left(x,y\right)e^{j\Phi \left(x,y\right)}$.

After filtering out the alternating component of the Fourier transform of I(x,y), c(x,y) is obtained with the inverse Fourier transform. Then the phase is retrieved from the following:

(14) $$\log c\left(x,y\right)=\log\left(\frac{1}{2}b\left(x,y\right)\right)+i\Phi \left(x,y\right)$$

With the above FFT method, the full field distribution of the fringe pattern obtained is shown in Figure 10b. 

Compared to the results calculated by the machine learning model and FFT, the phase distributions were similar, which established the accuracy of the measurement.

##### Complex Curved Surface

**Figure 11:**
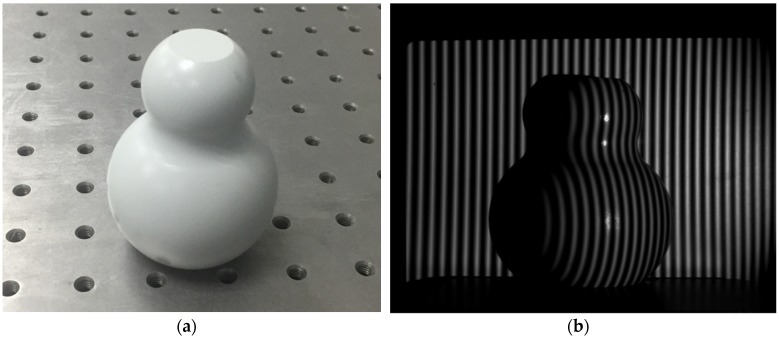
(**a**) Measured calabash; (**b**) measured calabash’s structured light fringe pattern.

Complex curved surfaces are common in the industry. In this study, we considered a calabash for performing the measurements. The measured calabash is shown in Figure 11a, and its structured light fringe pattern is shown in Figure 11b.

In this situation, the phase distribution on the *x*-axis was nonlinear. Therefore, the models were modified to fulfil this situation. Polynomial distributions were used to fit the nonlinear phase distributions. The models were trained by these samples:

Model 1: The training samples of the input intensity signals are defined as $X\left(x\right)=\cos\left(a_{i}x^{2}+b_{i}\cdot x+\varphi_{0i}^{\prime }\right)$, where the range of is from −5 to −1, the range of $b_{i}$ is from 9–26, the range of $\varphi_{0i}^{\prime }$ is from 0–1.7π, and the range of x is from 0–0.49. The training output samples include the corresponding phases $Y\left(x\right)=a_{i}x^{2}+b_{i}\cdot x+\varphi_{0i}^{\prime }$.

Model 2: The training samples of the input intensity signals are defined as $X\left(x\right)=\cos\left(a_{i}x^{2}+b_{i}\cdot x+\varphi_{0i}^{\prime }\right)$, where the range of is from −5 to −1, the range of $b_{i}$ is from 9–26, the range of $\varphi_{0i}^{\prime }$ is from 1.5π–2.5π, and the range of x is from 0–0.39. The training output samples include the corresponding phases $Y\left(x\right)=a_{i}x^{2}+b_{i}\cdot x+\varphi_{0i}^{\prime }$.

The specified interval of the initial phase for Model 1 and Model 2 were changed to [1.5, 4.7] and [4.7, 7.6], respectively.

**Figure 12:**
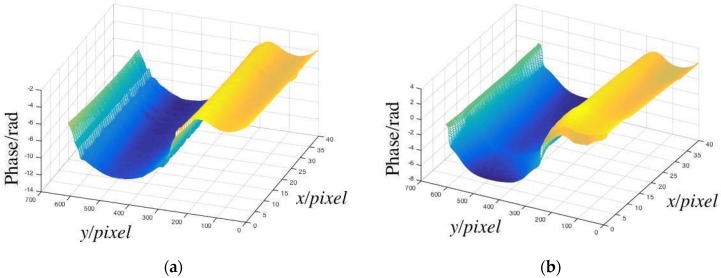
(**a**) Calabash’s phase distribution calculated by machine learning; (**b**) calabash’s phase distribution calculated by FFT.

The distributions of the fringe pattern calculated by the machine learning and FFT are shown in Figure 12a,b, respectively.

When the results calculated by the machine learning model were compared with those obtained from FFT, the phase distributions appeared similar, which established the accuracy of the measurement.

## 4. Discussion

It is observed from Figure 8 that the measurement error corresponding to columns 480–529 were under 1%, which validated the feasibility of the method we proposed. The surface measured in the experiment was a plane. In case the surface is not a plane, the samples trained by the machine learning model must be in a larger training set that includes some other light intensity signals with non-linear phases as the training samples. 

Some space was observed in Figure 7 without measuring points, which happens when one model is changed to another. As the light intensity signals were noisy, some phase differences were observed at the joint of the phase distributions recovered by the two models. In numerical analysis, the difference between the phase distributions of the same noise-free signal recovered by the two models could be ignored, as it was under 10^−3^ rad. Although the light intensity patterns were simply filtered by mean filtering, this filtration process must be improved in the future.

The training samples belonged to one dimension, and therefore, the results of the recovered phase distributions were unwrapped only in the dimension where the models were used. If the training samples belonged to two dimensions, the recovered phase distributions would be unwrapped in the both two dimensions. However, a more effective computing resource is required, if the training samples belong to two dimensions. Therefore, it is possible to use two-dimensional data for training, as long as adequate computing resources are available. In general, the model trained by one dimensional training samples is also sufficiently valid to complete the measurements.

## 5. Conclusions

In this study, the machine learning method was used to recover the phase of the structured light reflected from the object. Two models were trained by LSSVM to cover the initial phases from 0–2π. The phase distribution recovered by the machine learning models was unwrapped in the recovering dimension and wrapped only in the dimension that was perpendicular to the phase recovering dimension. Therefore, the phase distribution only needed to unwrap in one dimension, which is different from the traditional methods. The error in the measurement result was observed to be under 1%. Therefore, it was established that the structured light 3D measurement based on machine learning method is a precise measuring technology, which can be applied in several industries.
